# A distinct lipid metabolism signature of acute myeloid leukemia with prognostic value

**DOI:** 10.3389/fonc.2022.876981

**Published:** 2022-07-25

**Authors:** Ding Li, Jiaming Liang, Wei Yang, Wenbin Guo, Wenping Song, Wenzhou Zhang, Xuan Wu, Baoxia He

**Affiliations:** ^1^ Department of Pharmacy, The Affiliated Cancer Hospital of Zhengzhou University and Henan Cancer Hospital, Zhengzhou, China; ^2^ Department of Medicine, The Second Affiliated Hospital of Guangzhou Medical University, Guangzhou, China; ^3^ Department of Pathology, Pingtan Comprehensive Experimental Area Hospital, Fuzhou, China; ^4^ Department of Respiratory and Critical Care Medicine, Zhengzhou University People’s Hospital, Zhengzhou, China; ^5^ Academy of Medical Science, Zhengzhou University, Zhengzhou, China

**Keywords:** acute myeloid leukemia, prognosis, lipid metabolism, risk signature, immune landscape

## Abstract

**Background:**

Acute myeloid leukemia (AML) is a highly aggressive hematological malignancy characterized by extensive genetic abnormalities that might affect the prognosis and provide potential drug targets for treatment. Reprogramming of lipid metabolism plays important roles in tumorigenesis and progression and has been newly recognized a new hallmark of malignancy, and some related molecules in the signal pathways could be prognostic biomarkers and potential therapeutic targets for cancer treatment. However, the clinical value of lipid metabolism reprogramming in AML has not been systematically explored. In this study, we aim to explore the clinical value of lipid metabolism reprogramming and develop a prognostic risk signature for AML.

**Methods:**

We implemented univariate Cox regression analysis to identify the prognosis-related lipid metabolism genes, and then performed LASSO analysis to develop the risk signature with six lipid metabolism-related genes (LDLRAP1, PNPLA6, DGKA, PLA2G4A, CBR1, and EBP). The risk scores of samples were calculated and divided into low- and high-risk groups by the median risk score.

**Results:**

Survival analysis showed the high-risk group hold the significantly poorer outcomes than the low-risk group. The signature was validated in the GEO datasets and displayed a robust prognostic value in the stratification analysis. Multivariate analysis revealed the signature was an independent prognostic factor for AML patients and could serve as a potential prognostic biomarker in clinical evaluation. Furthermore, the risk signature was also found to be closely related to immune landscape and immunotherapy response in AML.

**Conclusions:**

Overall, we conducted a comprehensive analysis of lipid metabolism in AML and constructed a risk signature with six genes related to lipid metabolism for the malignancy, prognosis, and immune landscape of AML, and our study might contribute to better understanding in the use of metabolites and metabolic pathways as the potential prognostic biomarkers and therapeutic targets for AML.

## Background

Acute myeloid leukemia (AML) is a group of morphologically or genetically defined hematological malignancies with a poor prognosis (1). Relying on large scale mutational analysis in the genomic landscape, the treatment of AML improved significantly as the development of targeted therapies, such as targeting the FMS-like tyrosine kinase 3 (FLT3) and Isocitrate dehydrogenase (IDH) mutations, in the past several years (2). However, as a result of primary resistance to cytotoxic chemotherapy and high relapse rate, AML patients still face a dismal prognosis. AML is characterized by large-scale genomic changes and molecular mutations that affect the outcomes and provide potential therapeutic targets. And the risk stratification generating accurate prognosis and personalized therapeutic strategies are extremely important for cancer treatment. It is essential to identify new biomarkers to improve the risk stratification of AML, including those without cytogenetic alterations (3, 4).

Accumulating data has suggested that lipid metabolism is markedly reprogrammed in cancer cells, and lipid metabolism reprogramming plays critical roles in tumorigenesis and progression, including invasion, metastasis, and abnormal signaling (5). Moreover, lipid metabolism reprogramming show prognostic potential in clinical practice (6). Fatty acid synthase (FASN) which catalyzes the condensation of acetyl coenzyme A produces excess fatty acids to meet the demand for enough plasma membrane lipids in cell proliferation of cancer (7). And, high level of FASN expression is related to a poor outcome in patients with ovarian cancer (8). As a regulator of lipid metabolism, tumor protein D52 (TPD52) plays crucial roles in the formation of fatty acid storage and lipid droplets, and is a potential biomarker with prognostic value in various cancer types, including AML (9–11). Remarkably, as the key metabolic enzyme, IDH mutations are common genetic alterations found in more than 20% of AML patients and diversely affect the prognosis (12).

Consequently, given the biological role and therapeutic potential of lipid metabolism in AML, there is an urgent need to better understand the underlying molecular mechanisms of AML malignancy and to explore potential therapeutic targets for AML treatment using metabolic analysis. Here, we obtained the gene expression profile and corresponding clinical information of AML cohort from The Cancer Genome Atlas (TCGA) database to conduct a comprehensive analysis on the biological roles and prognostic value of the lipid metabolism-related genes in AML.

## Methods

### Data collection

The RNA-seq data and the relevant clinical information of AML patients are extracted from University of California, Santa Cruz (UCSC) Xena Browser (https://xenabrowser.net/). Subsequently, after excluding the samples with overall survival (OS) less than 30 days or missing clinical information, we included 144 samples for the analysis. Three other independent AML cohorts, GSE71014, GSE12417, and GSE37642, were extracted from Gene Expression Omnibus (https://www.ncbi.nlm.nih.gov/geo/) to validate the signature. Moreover, 26 lipid metabolism-related pathways including 1045 genes were extracted from the Molecular Signatures Database (MSigDB) (http://www.broad.mit.edu/gsea/msigdb/).

### Gene set variation analysis

GSVA was used to calculate the enrichment scores of AML patients in each lipid metabolism-related pathway, and then the samples were assigned into high-and low-score groups based on the median score. The correlation between the prognosis and enrichment score of each pathway in AML samples was analyzed to explore whether the identified pathways related to lipid metabolism was significantly associated with the prognosis.

### Generation of the prognostic signature using least absolute shrinkage and selection operator regularization

Survival analysis with the 1045 genes were performed to identify the potential prognosis-related genes for construction of the prognostic signature (P <0.05). Then, we performed LASSO analysis in the 163 candidate genes to compress variables with R package “glmnet”. As a type of linear regression, LASSO is a form of compression estimation that can emerge a more refined signature by setting up a penalty function, which could compress certain coefficients by defining others as zero, retaining the advantage of subset shrinkage. This is a type of processing used for bias estimation with complex collinearity data, and variables can be selected while estimating parameter, thereby solving the multicollinearity problem in regression analysis to a great extent. Finally, six lipid metabolism-related genes were included to construct the prognosis signature for AML.


$$\begin{array}{l} Risk\;Score=0.148491223769356*\text{EBP}+0.100012929390467*\text{CBR}1\\ \;+0.0974602543406011*\text{PLA}2\text{G}4\text{A}\;+\;0.0500714298035648\\ \;*\text{DGKA}+0.0278077820212605*\text{PNPLA}6\\ \;+0.00542436424230988*\text{LDLRAP}1 \end{array}$$

### Enrichment of functions and signaling pathways analysis

The samples were divided into high-risk group or the low-risk group by the median value of risk scores. Subsequently, R package “limma” was performed to identify differentially expressed genes (DEGs) with log2 (fold change)>1 and P<0.05 between the two risk groups. Functional enrichment analysis for the DEGs was based on Gene Ontology (GO) and Kyoto Encyclopedia of Genes and Genomes (KEGG) databases using R package “clusterPofolier” and determined which GO term or KEGG pathway is over-represented for specific gene set in which genes are dysregulated under certain conditions. P < 0.05 was considered as the significant pathways.

### Correlations of the signature and immune cell infiltration in AML

R package “GSVA” was used to perform GSVA which is designed to carry out a non-parametric unsupervised method to estimate the underlying molecule pathway activity based on RNA-seq data and gene expression microarray. The gene marker set, consisting of 782 genes that represent 28 immune cell types, is acquired from the previous research, and used to assess the infiltration of different immune cell types in the tumor microenvironment (TME). Then, the ssGSEA algorithm was executed to calculate the immune cell infiltration level in AML according to the gene expression profiles.

### Prediction of immunotherapy response

Tumor immune dysfunction and exclusion (TIDE) algorithm was performed to evaluate the response to immunotherapy of each sample using the gene expression profiles.

### Construction and evaluation of nomogram

To evaluate the clinically applicable potential of the signature to predict the OS of AML patients, we constructed a nomogram with clinical variables and signature score by through “rms” package. To evaluate the predictive performance of the nomogram, the “ROCsurvival” package was implemented to construct time-dependent ROC curves to estimate the 1-year, 3-year, and 5-year survival rate by the nomogram. Subsequently, “rms” package was executed to draw calibration curves to verify whether the predicted outcome showed good consistent with the practical.

### Survival analysis

Univariate analysis was performed to assess the correlation between the prognosis of AML patients and expression levels of lipid metabolic DEGs. The correlation between the prognosis of AML patients and risk score was also evaluated. The log-rank test and survival analysis were all performed using R package “survival”, while Kaplan-Meier curve was plotted using R package “surviminer”.

### Statistical analysis

Statistical analysis was carried out using the log-rank test for univariate Cox regression analysis. Pearson’s correlation analysis was performed to evaluate the association between the risk score and immune checkpoints, immune cell infiltration score and characteristic gene expression, respectively. Student’s t test was performed to assess the statistical significance among variables. And, P<0.05 was set as statistically significant. All statistical analysis were carried out in R version 4.0.2.

## Results

### Correlation between the prognosis and enrichment score of each pathway

First, we explored the correlation between the lipid metabolism and the prognosis of AML patients. As shown in **Supplemental Figure 1**, survival analysis indicated that the high-score group tended to be associated with a poor outcome compared with the low-score group, which revealed that the lipid metabolism-related pathways could be related to the prognosis.

### Construction of lipid metabolism-related gene prognostic signature

**Figure 1:**
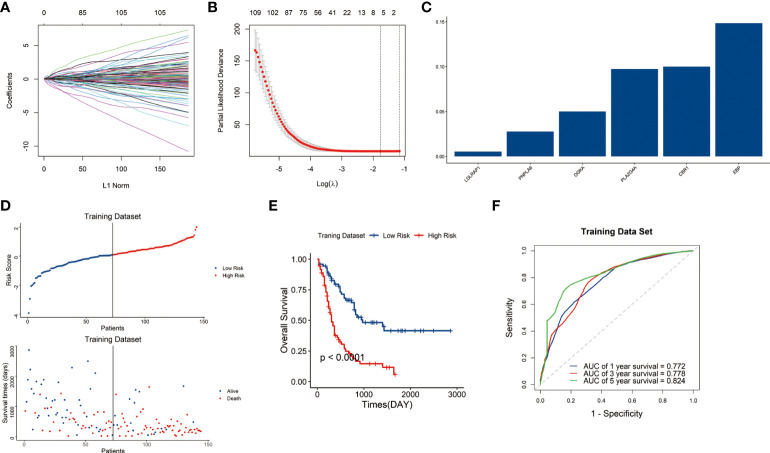
Construction of the lipid metabolism-related risk signature in the TCGA cohort. **(A)** Tuning parameters of OS-related genes to cross-verify the error curve. **(B)** Perpendicular imaginary lines to calculate the minimum criteria. **(C)** LASSO regression coefficient for each of six signature genes. **(D)** Distribution of the risk score and survival status of each sample. **(E)** Survival status of patients in the two risk groups classified by the signature. **(F)** ROC curves to predict the 1-, 3-, and 5-year survival.

Among the 1045 genes involved in the 26-lipid metabolism-related pathways, 163 lipid metabolism-related genes significantly related to prognosis (P<0.05) were identified from the 1045 genes through Pearson’s correlation analysis (**Figures 1A**, **B**). Then, six genes were finally identified through LASSO analysis. Subsequently, the lipid metabolism-related gene signature was constructed based on the expression levels of six genes LDLRAP1 (low-density lipoprotein receptor adaptor protein 1), PNPLA6 (patatin-like phospholipase domain containing 6), DGKA (diacylglycerol kinase alpha), PLA2G4A (phospholipase A2 group IVA), CBR1 (carbonyl reductase 1), and EBP (Emopamyl-binding protein) (**Figure 1C**).

The risk score of each sample in the training dataset was subsequently calculated with LASSO regression coefficients and expression levels of these six genes using formula mentioned above. Afterwards, the samples were assigned into high-risk and low-risk groups by the median risk score. Scatter plot showed the high-risk group was correlated with a higher mortality rate than the low-risk group (P<0.05) **(** **Figure 1D** **)**.

Kaplan–Meier curves indicated that the samples in high-risk group have significantly poorer outcomes than those in low-risk group (P<0.05) (**Figure 1E**). Then we conducted a time-dependent receiver operating characteristic (ROC) analysis to assess the predictive ability of the signature. The area under the curves (AUCs) of the 1-, 3-, and 5-year OS were 0.772, 0.778, and 0.824, respectively (**Figure 1F**). The results indicated the signature showed great sensitivity and specificity in predicting prognosis of AML patients.

### Validation of the prognostic signature using the GSE71014, GSE12417, and GSE37642

**Figure 2:**
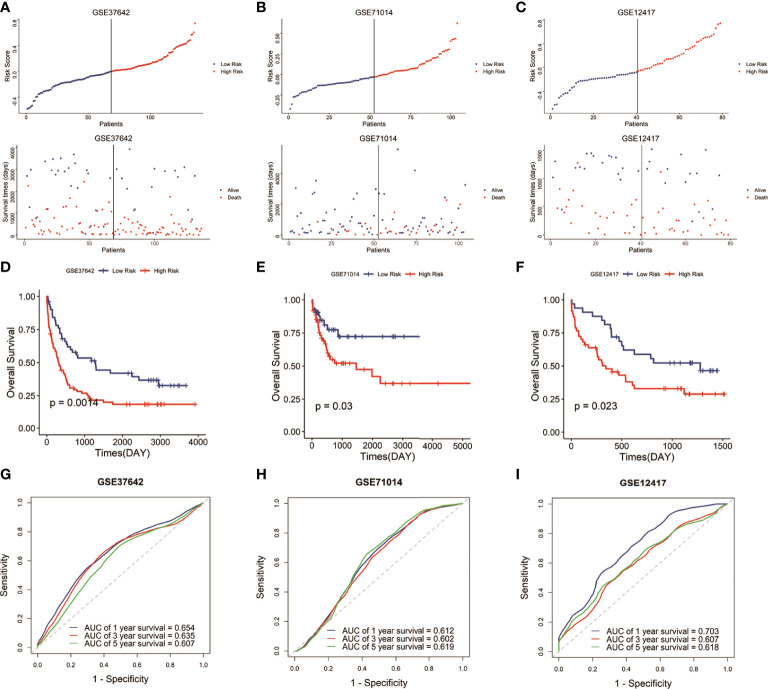
Validation of the signature in the GEO datasets. **(A–C)** Distribution of the risk score and survival status of each sample in the three validation cohorts. **(D–F)** Survival analysis of the two risk groups classified by the signature in the three testing datasets. **(G–I)** ROC curves to predict the 1-, 3-, and 5-year survival in the three testing datasets.

To verify the predictive reliability and applicability of the signature, we then calculated the risk score of each sample in the other three AML cohorts (GSE37642, GSE71014, and GSE12417) based on the same formula and assigned the samples into low-risk and high-risk groups by the median risk score in each cohort. As expected, in the three validation cohorts, the high-risk groups were associated with a high mortality rate compared with the low-risk groups (**Figures 2A**–**C**). And the high-risk groups demonstrated lower survival time than the low-risk groups (**Figures 2D**–**F**). The ROC curves in the three testing cohorts demonstrated the signature present a robust and stable predictive ability in the prognosis of AML patients, and the AUCs to predict 1-, 3-, and 5-year OS were 0.654, 0.635, and 0.607 (GSE37642 cohort), 0.612, 0.602, and 0.619 (GSE71014 cohort), and 0.703, 0.604, and 0.618 (GSE12417 cohort), respectively (**Figures 2G**–**I**). Overall, the results demonstrated that the six-gene signature had a stable and robust predictive power in AML.

### Correlation of the risk signature with clinical variables

**Figure 3:**
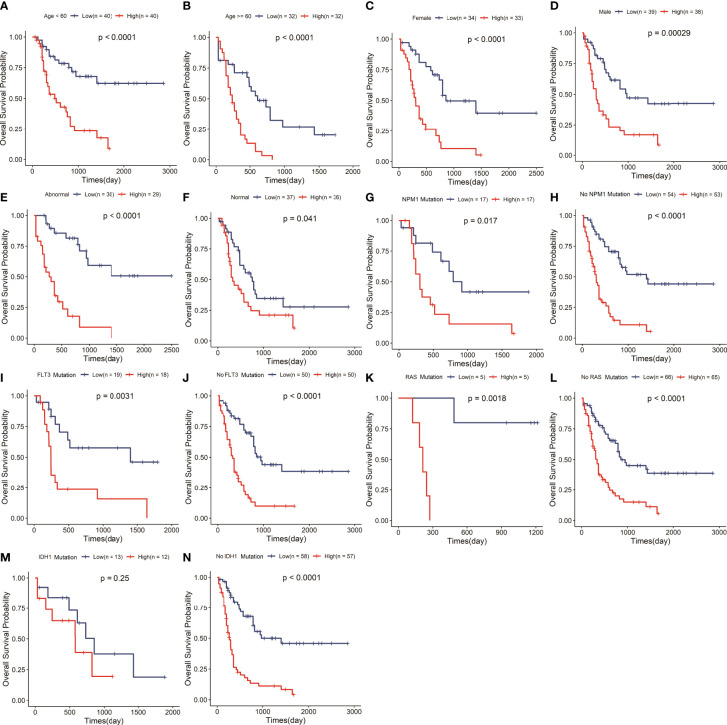
The signature retained great prognostic value in different subgroups of AML patients. Survival analysis in the two risk groups under diverse situations classified by several clinical features including age **(A, B)**, gender **(C, D)**, status of chromosome **(E, F)**, status of NPM1 **(G, H)**, status of FLT3 **(I, J)**, status of RAS **(K, L)** and status of IDH1 **(M, N)**.

To explore whether the risk signature is an independent prognostic factor in AML, we carried out a stratification analysis to assess the efficiency of the signature in various subgroups with different clinical variables, including age, gender, chromosomal abnormality or not, FLT3, IDH1, NPM1 and RAS mutation status (**Supplemental Table 1**). The results of survival analysis that the low-risk group hold longer OS than the high-risk group in the aged<60 or≥60 years subgroup (P<0.05) (**Figures 3A**, **B**), female or male subgroup (P<0.05) (**Figures 3C**, **D**), chromosomal abnormality or not subgroup (P<0.05) (**Figures 3E**, **F**), mutant or wildtype NPM1 subgroup (P<0.05) (**Figures 3G**, **H**), mutant or wildtype FLT3 subgroup (P<0.05) (**Figures 3I**, **J**), and mutant or wildtype RAS subgroup (P<0.05) (**Figures 3K**, **L**). While the low-risk group had a significantly longer OS in wildtype IDH1 subgroup, there was no significant difference in OS between the two risk groups with IDH1 mutation (**Figures 3M**, **N**). Stratification analysis showed that signature had great efficiency and keep stable in diverse situations.

### Construction and validation of the signature based on nomogram

**Figure 4:**
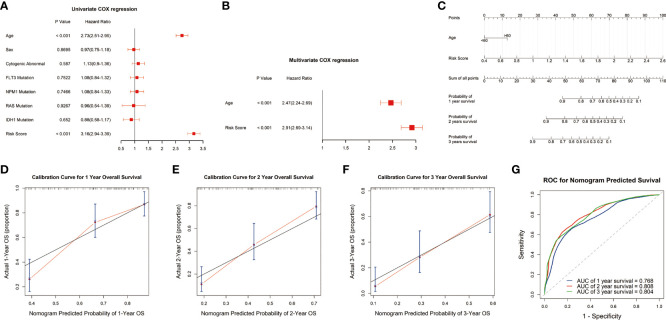
Construction and validation of the nomogram. **(A, B)** Univariate and multivariate analyses showed that risk score was an independent prognostic factor in the TCGA cohort. **(C)** Nomogram consisting of risk score and age. **(D-F)** Calibration plots of the nomogram to predict the probability of survival and actual survival rates at 1-, 2-, and 3-year in the TCGA cohort. **(G)** ROC curve to predict 1-, 2-, and 3-year survival for the nomogram in the TCGA dataset.

To evaluate the potential clinical application of the risk signature, we constructed a nomogram to predict the 1-, 2-, and 3-year survival. The univariate analysis showed that the risk score and age were closely correlated with survival outcome of AML patients (P<0.05) (**Figure 4A**). Multivariate analysis revealed that the risk score presented an independent prognostic factor after adjusting for these clinical variables, although age was also independent (P<0.05) (**Figure 4B**). Then, we developed a nomogram based on the age and risk score with clinical accessibility and independent prognostic ability (**Figure 4C**). The Calibration plots showed that the nomogram had ideal concordance between the predictive and practical survival rates at 1-, 2-, and 3-year (**Figures 4D**–**F**). In addition, the ROC curves showed the nomogram hold favorable accuracy in estimating the 1-, 2-, and 3-year survival of AML patients. And, the AUCs were 0.768, 0.808, and 0.804, which revealed that the nomogram had a favorable capability to estimate the survival for AML patients (**Figure 4G**).

### Functional enrichment analysis

**Figure 5:**
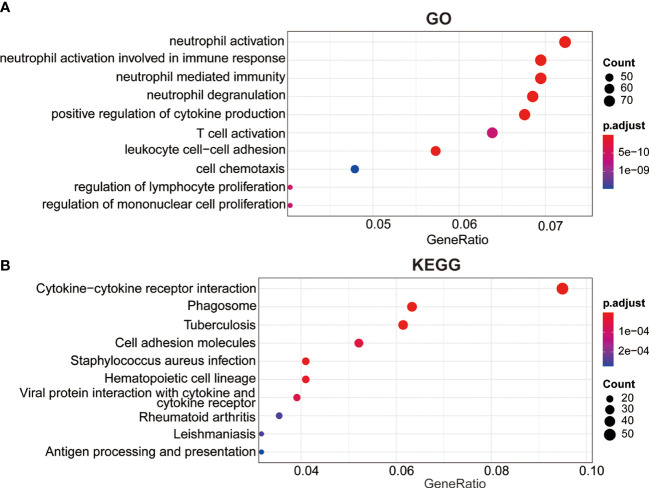
Cellular biological effects related to the signature. **(A)** Gene ontology (GO) biological process analysis. **(B)** Kyoto Encyclopedia of Genes and Genomes (KEGG) pathway analysis.

To further investigate underlying mechanism of this prognosis relevance, we performed GO and KEGG analysis based on the DEGs between the two risk groups. The GO analysis revealed these DEGs were primarily enriched in these terms, including positive regulation of cytokine production, neutrophil activation, T cell activation, neutrophil degranulation, neutrophil mediated immunity, leukocyte cell-cell adhesion (**Figure 5A**). The KEGG analysis revealed that these genes were involved in cytokine-cytokine receptor interaction, phagosome, cell adhesion molecules, antigen processing and presentation and hematopoietic cell lineage pathways, which might provide us with insights into cancer progression and potential molecular mechanisms (**Figure 5B**).

### Immune cell infiltration of high-risk and low-risk groups with AML

**Figure 6:**
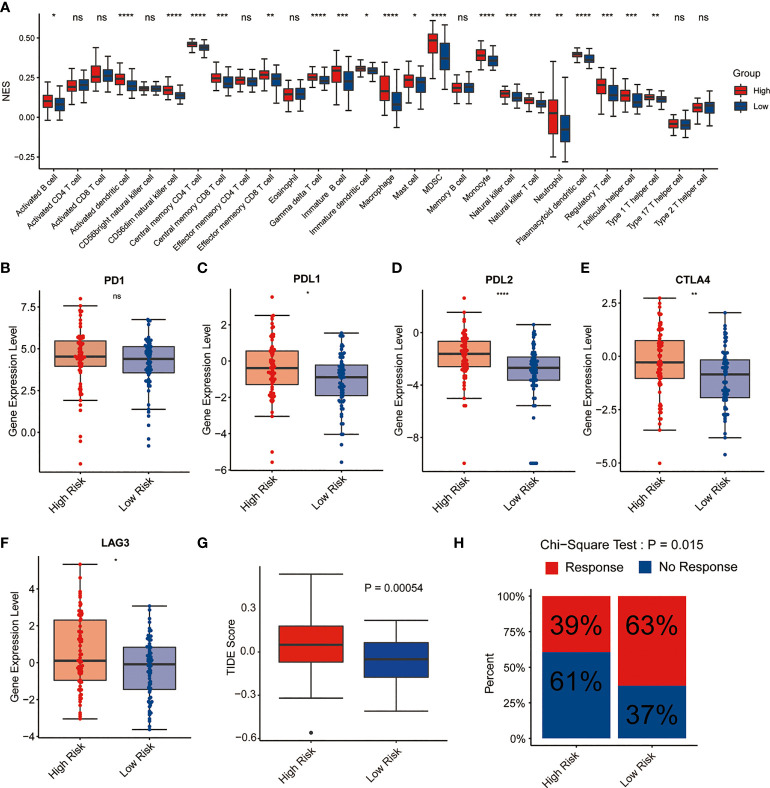
Immune cell infiltration and immunotherapy response of high-risk and low-risk groups with AML. **(A)** Correlation of risk score and immune cell infiltration by ssGSEA algorithm. **(B–F)** Expression of PD1, PDL1, PDL2, CTLA4, and LAG3 in the two risk groups. **(G)** TIDE score in the two risk groups. **(H)** Immunotherapy response rate in the two risk groups. *P<0.05, ** P<0.01, *** P<0.001, ****P<0.0001, ns, not significant.

Since the neutrophil activation and T cell activation were enriched in GO analysis, we next explore the potential relationship between the signature and TME as well as immune response. CIBERSORT algorithm was performed to assess the immune cell infiltrating level in TME involved in the signature. The results demonstrated that high-risk AML patients had significantly high fractions in dendritic cell, memory CD4+, CD8+ T cell, immature B cells, macrophages, MDSC, monocyte, NK cells, and regulatory T cells compared with low-risk ones. However, no significant difference was observed in the activated CD4+, CD8+ T cell, and CD56 bright NK cell between the two risk groups, suggesting this lipid metabolism signature might be highly associated with an immunosuppressive TME (**Figure 6A**). Therefore, the expression levels of immunosuppressive genes between the two risk groups were further explored. As shown in **Figures 6B**–**F**, the common immune checkpoints, including PDL1, PDL2, CTLA4, and LAG3, were significantly upregulated in the high-risk group. Then, we compared the difference in the immune evasion and immunotherapy response. And the results revealed the low-risk group has a lower immune evasion score and higher immunotherapy response rate than the high-risk group (**Figures 6G**–**H**).

## Discussion

AML is an uncommon but potentially catastrophic heterogeneous hematologic malignancy characterized by the malignant proliferations of myeloid blasts or progranulocytes instead of normal differentiation (1). Despite advances in understanding mutational heterogeneity in leukemogenesis and disease progression have led to novel targeted agents developed for AML, the prognosis of AML patients remains unsatisfied. The identification of new molecular biomarkers contributes to new understanding of the molecular basis of AML, and is significantly useful in diagnostic and disease classifications, new target agent development, as well as predicting response to treatment (4).

The transformation from normal cells into malignant cells accompanies multiple biological characteristics, among which metabolic reprogramming is one of the most prominent, and contributes to promote tumorigenesis and tumor progression (13). Despite AML has been studied thoroughly at the cellular and molecular level, including genetic, epigenetic, and protein, a comprehensive and systematic metabolic signature for this group of disease have been less studied. In this study, we aimed to construct a risk signature to stratify AML patients based on the lipid metabolic landscape. The six-gene signature with stable predictive performance was constructed by univariate Cox regression analysis and LASSO, and was significantly related to clinical outcomes and immune cell infiltration.

Interestingly, these six genes involved in the signature had been reported to contribute to promote pathogenesis and disease progression in various diseases and cancer types. Previous study found that the common PLA2G4A upregulation in cancer cells contributes to their migration and invasion and is significantly correlated with unfavorable prognosis (14, 15). PLA2G4A overexpression contributed to the malignant phenotype of AML cells together with its partners and is correlated to a poor prognosis in the patients with non-M3/NPM1 wildtype or Hoxa9- and Meis1-dependent AML (16, 17). DGKA is the family member of conserved membrane lipid kinases and has been involved in human cancers (18–20). DGKA could enhance hepatocellular carcinoma progression *via* Ras-Raf-MEK-ERK pathway and promote platinum resistance by activating c-JUN-WEE1 signaling in ovarian cancer (21, 22). CBR1 plays a critical role in tumor metastasis and growth and its expression level is elevated in cancer tissues compared with paired normal lung tissue (23–25). Furthermore, inhibition of CBR1 safely improves the efficacy of doxorubicin in breast cancer treatment (26). EBP is the essential enzyme required for cholesterol biosynthesis, and inhibition of EBP led to cancer cell death *via* depletion of downstream sterols (27).

However, the other two genes, LDLRAP1 and PNPLA6, have not been found to be related to cancer patients’ prognosis at present. Previous evidence indicated that LDLRAP1 dysregulation leads to low-density lipoprotein receptors on the apical surface, and reduce low-density lipoprotein uptake, thereby decreasing LDL-cholesterol metabolism, which could cause familial hypercholesterolemia (28). PNPLA6 mutations play crucial roles in the central nervous system disorders (29). This study was the first to find that LDLRAP1 and PNPLA6 could serve as prognostic markers of AML.

Our study suggested that this signature could precisely stratify AML patients into different risk groups. The Kaplan-Meier curve showed that risk score divided the patients independent of present genetic aberrations besides IDH+ mutation (**Figure 3**). IDH1 is an important enzyme involved in multiple metabolic (especially the lipid and fatty acid synthesis) and epigenetic cellular processes, which lead to abnormal DNA and histone methylation, increased cell proliferation, altered gene expression and leukemogenesis. IDH1 mutations were associated with worse OS, event-free survival and complete remission of AML patients. It is suggested that the AML patients with IDH1 mutation often suffered a poor outcome, which maybe result in no statistically significant difference in survival between high and low risk groups. Moreover, the impact of IDH1 mutation on lipid metabolism maybe interfere with the predictive value of our signature related to lipid metabolism, which also verifies the important role of lipid metabolism on the prognosis of AML from the side. While, IDH1 mutations exist in only 7~14% of AML patients, our risk model has great efficiency and keep stable in the vast majority situations.

GO and KEGG revealed that the functions of DEGs between high- and low-risk groups were mainly enriched in immune-related pathways, such as T cell activation, neutrophil activation, cytokine production, and cytokine-cytokine receptor interaction (**Figure 5**), indicating that lipid metabolism may affect the outcome of AML patients *via* some immune mechanisms. Here, the analysis of immune cell infiltration in TME showed that this lipid metabolism signature might be highly associated with an immunosuppressive TME (**Figure 6A**). Immune checkpoints play major roles in carcinogenesis and progression by through enhancing tumor immunosuppression (30). In this study, the common immune checkpoints, including PDL1/2, LAG3, and CTLA4, were significantly upregulated in the high-risk AML population, which indicated an immunosuppressive TME of bone marrow in the high risk group (**Figures 6B**–**F**). Immunotherapy resistance has been reported in most leukemia patients, partially due to the immunosuppressive TME in bone marrow. Furthermore, leukemia cells could generate the immunosuppressive TME in bone marrow by through reprogramming the metabolism to produce enough energy and to escape immune surveillance. In addition, leukemia cells could also escape immune surveillance by through expressing immune checkpoint molecules (30). These above evidences support the results of our study which showed the low-risk group has a lower immune evasion score and higher immunotherapy response rate than the high-risk group (**Figures 6G**–**H**).

We conducted a pan-cancer analysis of the signature to explore whether the signature had a predictive value in other cancers. The risk score of each sample in different cancer type was calculated with the same signature forum and assigned to high- and low-risk groups. Similarly, the survival analysis demonstrated that the high-risk group hold a significantly poor outcome compared with the low-risk group in adrenocortical carcinoma, colon adenocarcinoma, renal clear cell carcinoma, head and neck squamous cell carcinoma, lower-grade glioma, uterine corpus endometrial carcinoma, and uveal melanoma cohort from TCGA database. However, there are no significant difference in OS between the two risk groups of other 25 cancer types (**Supplemental Figure 2**).

Furthermore, we also analyzed the correlation between the prognosis and each gene in the signature, respectively. The survival analysis showed that each gene from the signature was significantly associated with the poor prognosis of AML patients, and the differential expression analysis demonstrated all of these six genes were over-expressed in the high-risk group, which enhance the accuracy of the signature in predicting the prognosis (**Supplemental Figures 3**, **4**).

Metabolic reprogramming plays important roles in cancer initiation and progression. However, thorough understanding of lipid metabolism in AML is still limited. The prognostic prediction of AML highlights an urgent need for other non-invasive diagnostics, such as circulating metabolic biomarkers and molecular test, to enhance the screening efficiency, avoid over-treatment, decrease costs, and improve clinical outcomes (31). Strikingly, this study generated a biomarker panel of six lipid metabolism-related genes was valuable in classifying AML into different risk groups. Our findings revealed the application of lipid metabolomics for potential biomarker discovery, thus serving as the screen for the at-risk subgroups. In addition, therapeutic agents targeting such key metabolic pathways could play important roles in the management of AML patients, which need to be intensively studied in future work.

The strength of this study is that we performed a systematically analysis based on the public database for the first time, and explored the clinical application of lipid metabolism in the AML. Although this experiment is based on a large sample of omics data, there are some limitations in our study. Firstly, the RNA-seq data and clinical information are extracted from the public database. Secondly, the mechanism how the included lipid metabolism-related genes modulate the precise process of AML is unclear. Lastly, the prognostic signature is based primarily on bioinformatics analysis and needs to be verified in a large-scale and multicenter clinical cohort.

## Conclusions

We conducted a comprehensive analysis of lipid metabolism in AML and constructed a risk signature with six genes related to lipid metabolism for the malignancy, prognosis, and immune landscape of AML, and our study might contribute to better understanding in the use of metabolites and metabolic pathways as the potential prognostic biomarkers and therapeutic targets for AML.

## Data availability statement

Publicly available datasets were analyzed in this study. This data can be found here: https://www.ncbi.nlm.nih.gov/geo/, GSE37642; GSE71014; and GSE12417.

## Author contributions

XW, DL, WZZ and BXH designed the research. JML and WBG analyzed the data. DL and XW wrote the paper with contributions from WY and WPS. All authors have read and approved the manuscript.

## Funding

This study was supported by Henan Provincial Science and Technology Research Project (202102310157), Medical Science and Technology Research Plan (Joint Construction) Project of Henan Province (LHGJ20190676).

## Acknowledgments

We are grateful to the contributors to the public databases used in this study.

## Conflict of interest

The authors declare that the research was conducted in the absence of any commercial or financial relationships that could be construed as a potential conflict of interest.

## Publisher’s note

All claims expressed in this article are solely those of the authors and do not necessarily represent those of their affiliated organizations, or those of the publisher, the editors and the reviewers. Any product that may be evaluated in this article, or claim that may be made by its manufacturer, is not guaranteed or endorsed by the publisher.

## Supplementary material

Supplemental Figure 1 Correlation of each lipid metabolism-related pathway and prognosis. Survival analysis in the two subgroups of patients classified by the enrichment score of each lipid metabolism-related pathway.

Supplemental Figure 2 The predictive value of the signature in other cancers. Survival analysis in the two risk groups based on the signature in different types of cancers.

Supplemental Figure 3 Correlation of each signature gene and prognosis. Survival analysis in the two subgroups of patients classified by the expression level of each gene in the signature.

Supplemental Figure 4 The expression levels of these six genes in patient samples from TCGA.

Supplemental Table 1 The clinical characteristics for AML cases in TCGA.

## Abbreviations

AML, acute myelogenous leukemia; GO, Gene Ontology; KEGG, Kyoto Encyclopedia of Genes and Genomes; DEGs, differentially expressed genes; OS, overall survival; TCGA, The Cancer Genome Atlas; TIDE, tumor immune dysfunction and exclusion; GSVA, Gene Set Variation Analysis; LASSO, least absolute shrinkage and selection operator; ROC, receiver operating characteristic; FLT3, FMS-like tyrosine kinase 3; IDH, isocitrate dehydrogenase; FASN, fatty acid synthase; TPD52, tumor protein D52; LDLRAP1, low-density lipoprotein receptor adaptor protein 1; PNPLA6, patatin-like phospholipase domain containing 6; DGKA, diacylglycerol kinase alpha; PLA2G4A, phospholipase A2 group IVA; CBR1, carbonyl reductase 1; EBP, Emopamyl-binding protein; PD1, programmed cell death protein 1; PDL1/2, Programmed cell death 1 ligand 1/2; CTLA4, cytotoxic T-lymphocyte-associated protein 4; LAG3, Lymphocyte Activation Gene-3.
