# Supplementary figures and images for: A distinct lipid metabolism signature of acute myeloid leukemia with prognostic value
Source: Front Oncol. 2022 Jul 25;12:876981. doi: 10.3389/fonc.2022.876981 (PMC9359125; doi:10.3389/fonc.2022.876981)

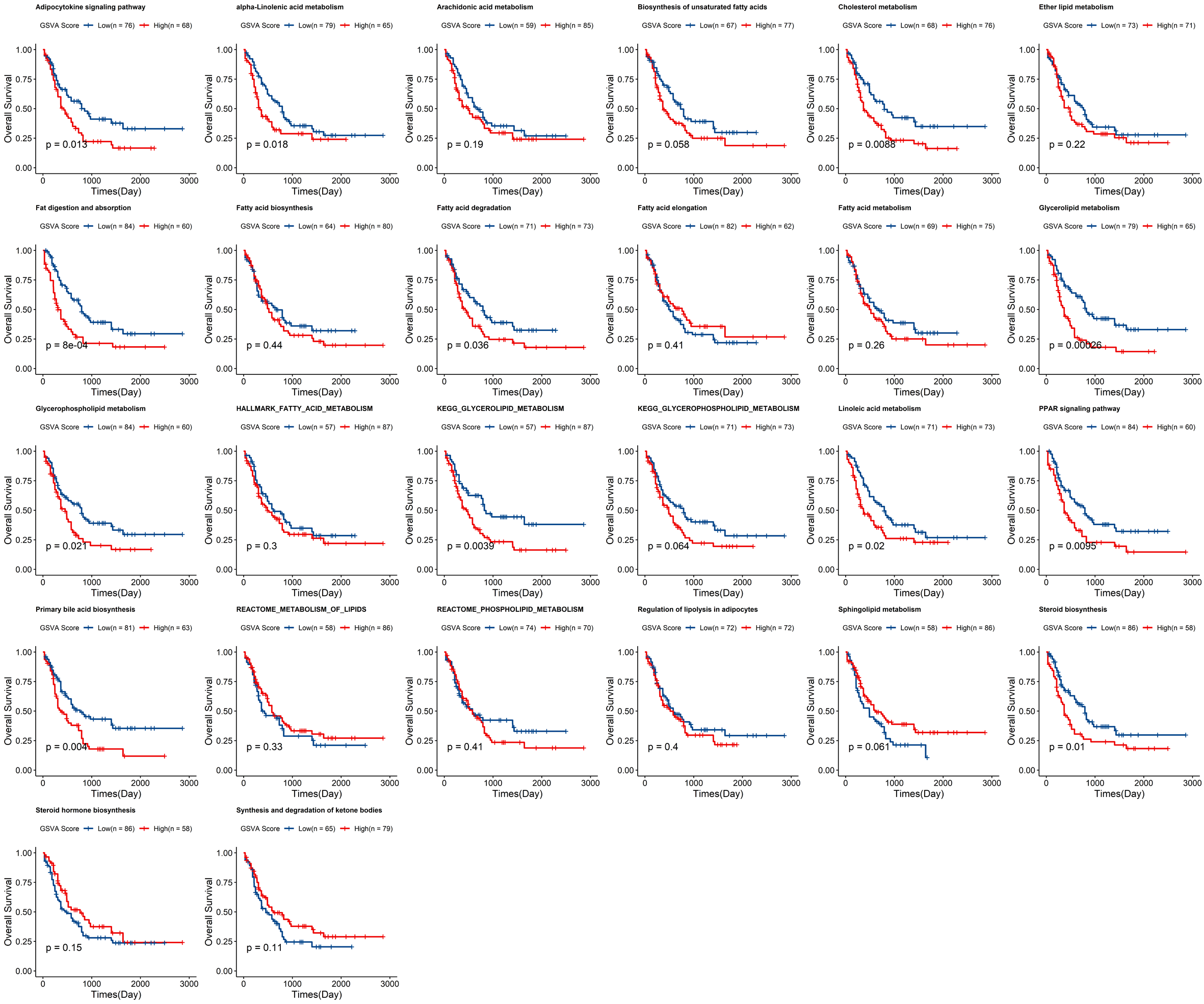

Supplement: Supplemental Figure 1 — Correlation of each lipid metabolism-related pathway and prognosis. Survival analysis in the two subgroups of patients classified by the enrichment score of each lipid metabolism-related pathway. [file Image_1.tif]

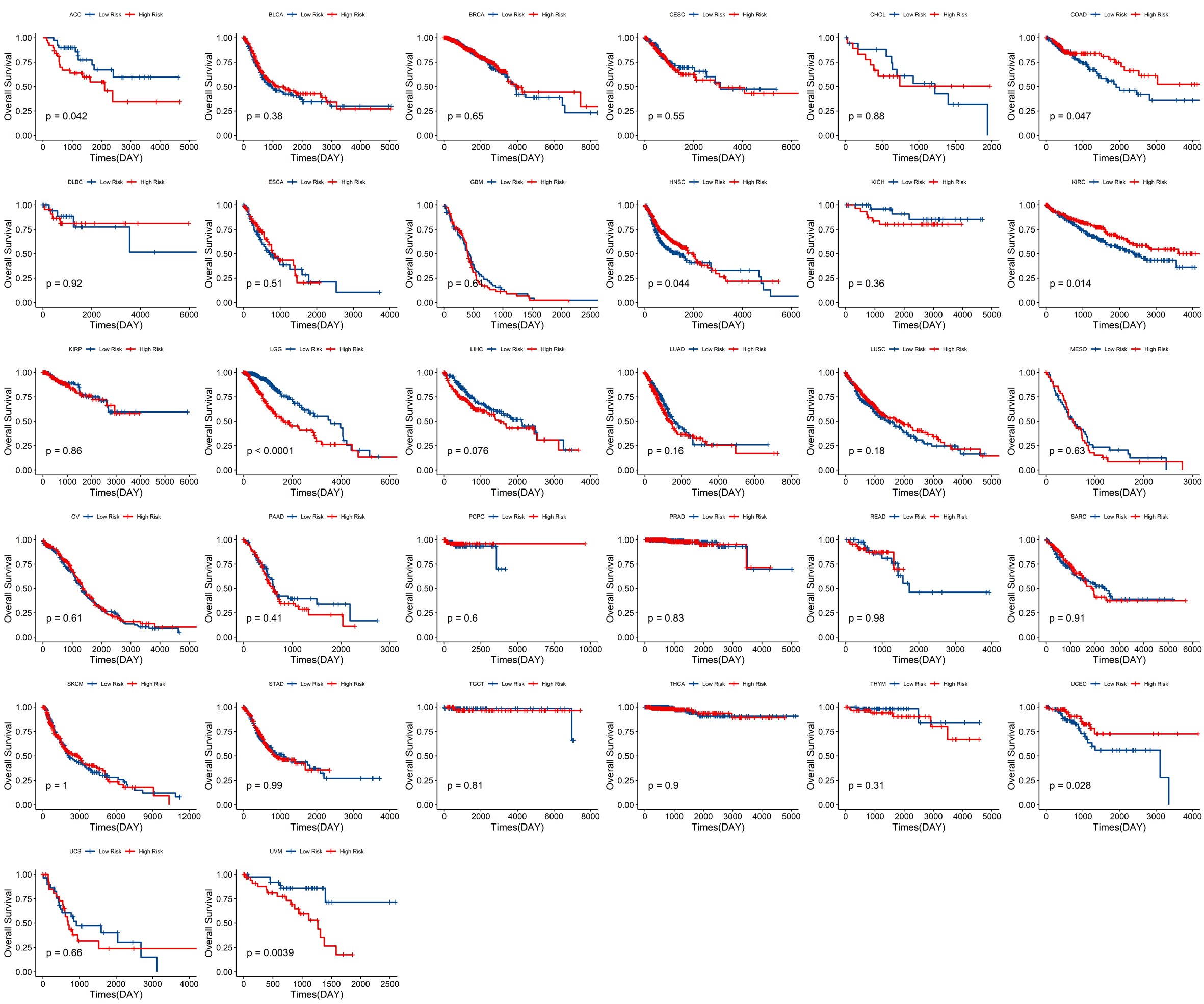

Supplement: Supplemental Figure 2 — The predictive value of the signature in other cancers. Survival analysis in the two risk groups based on the signature in different types of cancers. [file Image_2.tif]

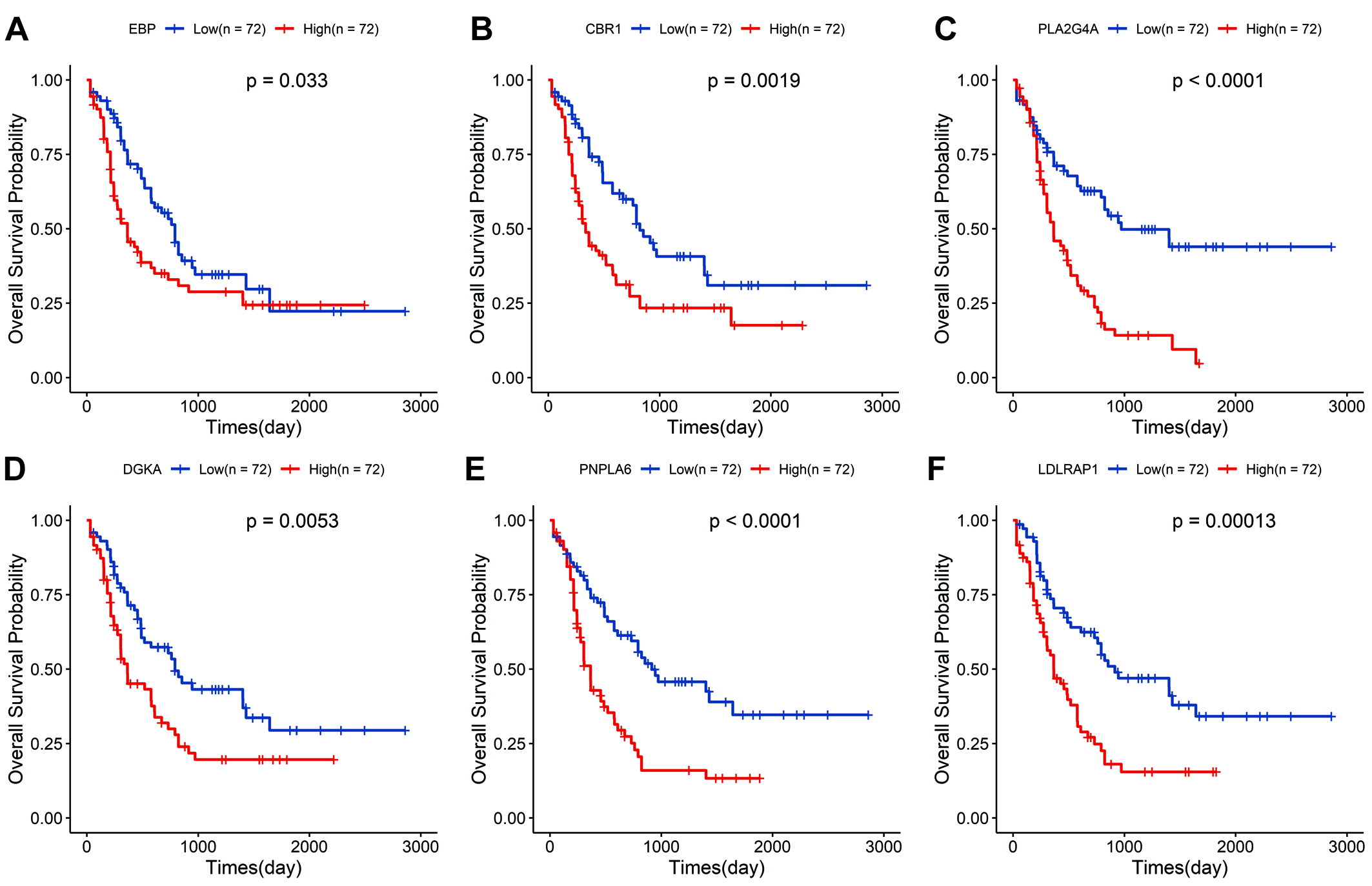

Supplement: Supplemental Figure 3 — Correlation of each signature gene and prognosis. Survival analysis in the two subgroups of patients classified by the expression level of each gene in the signature. [file Image_3.tif]

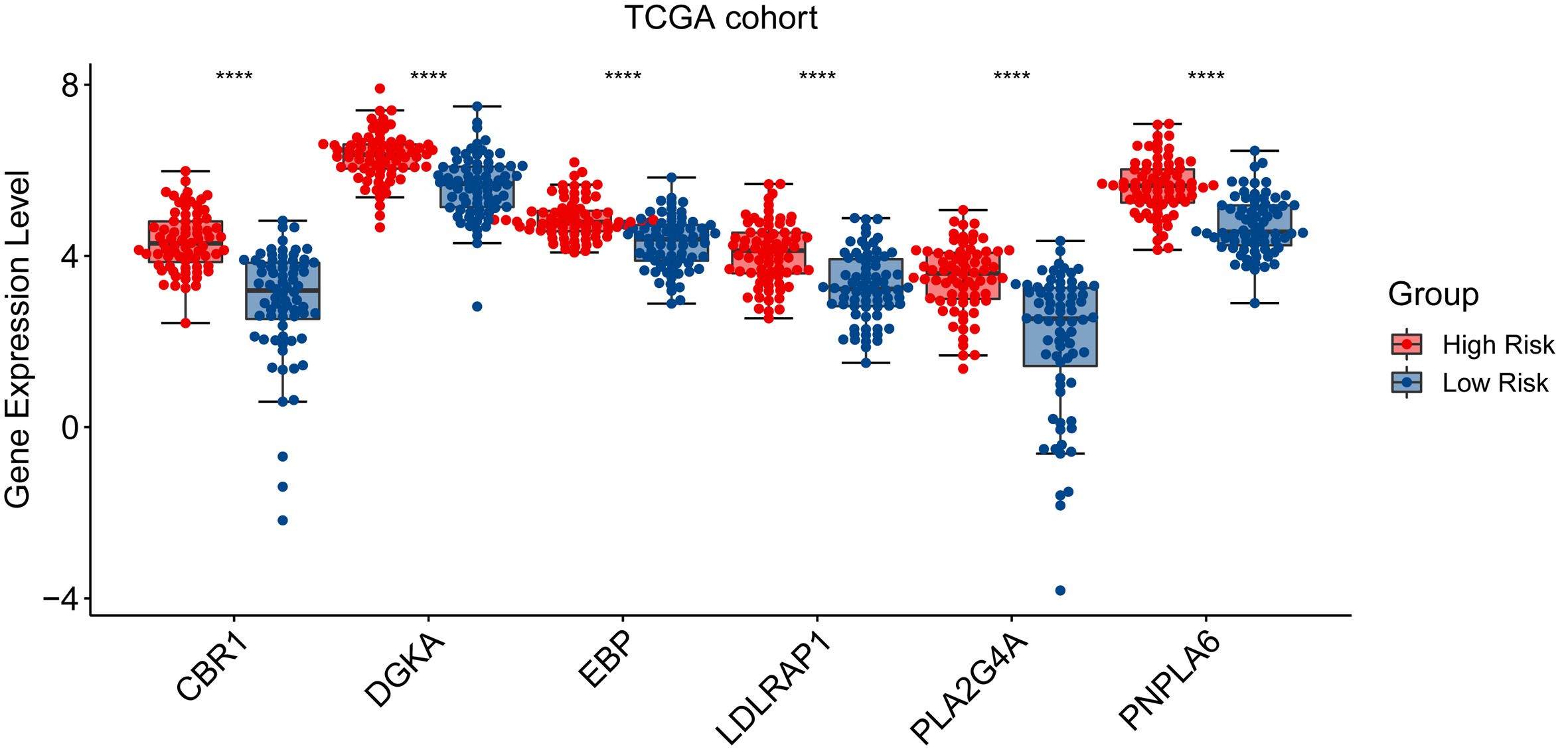

Supplement: Supplemental Figure 4 — The expression levels of these six genes in patient samples from TCGA. [file Image_4.tif]
