# Supplementary material for: A distinct lipid metabolism signature of acute myeloid leukemia with prognostic value
Source: Front Oncol. 2022 Jul 25;12:876981. doi: 10.3389/fonc.2022.876981 (PMC9359125; doi:10.3389/fonc.2022.876981)
Supplement: Supplemental Table 1 — The clinical characteristics for AML cases in TCGA. [file Table_1.docx]

| Variable | N | low, N = 72^1^ | high, N = 72^1^ | p-value^2^ |
| --- | --- | --- | --- | --- |
| **Age** | 144 | 51 (38, 61) | 60 (51, 69) | <0.001 |
| **Sex** | 144 |  |  | 0.13 |
| Female |  | 38 (53%) | 29 (40%) |  |
| Male |  | 34 (47%) | 43 (60%) |  |
| **Chromosome** | 132 |  |  | 0.2 |
| Abnormal |  | 33 (50%) | 26 (39%) |  |
| Normal |  | 33 (50%) | 40 (61%) |  |
| **FLT3** | 137 |  |  | 0.3 |
| Negative |  | 53 (77%) | 47 (69%) |  |
| Positive |  | 16 (23%) | 21 (31%) |  |
| **NPM1** | 141 |  |  | 0.2 |
| Negative |  | 58 (81%) | 49 (71%) |  |
| Positive |  | 14 (19%) | 20 (29%) |  |
| **RAS** | 141 |  |  | >0.9 |
| Negative |  | 67 (93%) | 64 (93%) |  |
| Positive |  | 5 (6.9%) | 5 (7.2%) |  |
| **IDH1** | 140 |  |  | 0.6 |
| Negative |  | 57 (80%) | 58 (84%) |  |
| Positive |  | 14 (20%) | 11 (16%) |  |
| ^1^Median (IQR); n (%) | | | | |
| ^2^Wilcoxon rank sum test; Pearson's Chi-squared test | | | | |
